# Low CD4/CD8 Ratio in Bronchus-Associated Lymphoid Tissue Is Associated with Lung Allograft Rejection

**DOI:** 10.1155/2012/928081

**Published:** 2012-08-08

**Authors:** K. V. Shenoy, C. Solomides, F. Cordova, T. J. Rogers, D. Ciccolella, G. J. Criner

**Affiliations:** Division of Pulmonary and Critical Care Medicine, Temple University School of Medicine, Philadelphia, PA 19140, USA

## Abstract

*Background*. Bronchus-associated lymphoid tissue (BALT) has been associated with lung allograft rejection in rat transplant models. In human transplant recipients, BALT has not been linked to clinically significant rejection. We hypothesize that the immunohistochemical composition of BALT varies with the presence of acute lung allograft rejection. *Methods*. We retrospectively examined 40 human lung allograft recipients transplanted from 3/1/1999 to 6/1/2008. Patients were grouped by frequency and severity of acute rejection based on International Society of Heart Lung Transplant (ISHLT) criteria. Transbronchial biopsies were reviewed for BALT by a blinded pathologist. BALT if present was immunohistochemically stained to determine T-and B-cell subpopulations. *Results*. BALT presence was associated with an increased frequency of acute rejection episodes in the first year after transplantation. Patients with a lower CD4/CD8 ratio had an increased rejection rate; however, BALT size or densities of T-cell and B-cell subpopulations did not correlate with rejection rate. *Conclusion*. The presence of BALT is associated with an increased frequency of rejection one year after transplant. The lower the CD4/CD8 ratio, the more acute rejection episodes occur in the first year after transplantation. The immunohistochemical composition of BALT may predict patients prone to frequent episodes of acute cellular rejection.

## 1. Introduction

Bronchus-associated lymphoid tissue (BALT) presence has been described in many animal species and in normal human lung tissue. BALT consists of follicular lymphoid aggregates within the mucosa of the bronchial tree [1]. BALT plays a role in local host defense similar to gut associated lymphoid tissue and tonsils. In addition, it is involved in antigen distribution and processing between the lung tissue and the mediastinal lymph nodes. In humans with inflammatory disease such as chronic obstructive pulmonary disease (COPD) and rheumatologic lung diseases the presence and number of BALT increases as the severity of disease worsens [2, 3]. Specifically there are an increased number of lymphoid follicles as the degree of airflow obstruction worsens in COPD [2]. In diseases such as rheumatoid lung, the presence of well-organized BALT is associated with the local expression of cytokines and enzymes that play a role in the pathobiology of autoimmune lung diseases [3]. BALT is often seen on transbronchial biopsy (TBBx) specimens in lung transplantation; however, the significance of its presence is not well understood. In rat transplantation models, the greater number of BALT lymphocytes accelerates acute cellular rejection and the elimination of BALT through irradiation of the donor rat prior to transplantation abrogates rejection [4]. In human lung transplantation, the detection of BALT was associated with low-grade or no rejection (AO or A1) by International Society of Heart Lung Transplantation (ISHLT) criteria [5]. This led to the speculation that BALT in human lung allografts might be involved in immunological tolerance.

The pathobiology of acute cellular rejection shows an increased number of perivascular T cells, with increased numbers of cytotoxic T cells [6]. Given the known immunohistochemical pattern of acute rejection, we propose that BALT found in those with multiple episodes of rejection is immunohistochemically different from BALT in those with minimal rejection. Specifically, we hypothesize that multiple rejections leads to an increase in the accumulation of CD8 T cells in the BALT tissue.

## 2. Materials and Methods 

### 2.1. Patients

We retrospectively examined forty human lung allograft recipients from 3/01/1999 to 6/01/2008. Patients were chosen by frequency and severity of acute rejection based on International Society of Heart Lung Transplant (ISHLT) criteria [7]. The multiple rejection group was defined as having three or more episodes of A2 or higher rejection within the first year after transplantation. The minimal rejection group was defined as having one (A2 or lower) or no rejections of within the first year after transplantation. All patients received standard immunosuppression with a calcineurin inhibitor, cell cycle inhibitor, and corticosteroids. Most patients received induction therapy with basiliximab at release of cross clamp and on postoperative day 4. Either tacrolimus or cyclosporine was administered starting on postoperative day 1 with goal trough levels of 350–400 ng/mL or 8–10 ng/dL, respectively, one year after transplantation. Mycophenolate or azathioprine was administered one week after transplantation and titrated to goal of 1 gram every 12 hours or 150 mg daily, respectively. One gram of methylprednisolone was administered at release of cross clamp and then tapered to a goal of 0.1 mg/kg/day of prednisone by three months. These medications were adjusted based on side effects, white blood cell, and platelet counts. Patients underwent surveillance TBBx at 1 month, 2 months, 4 months, 6 months and 12 months after transplant. Also TBBx was performed based on respiratory symptoms or decline in spirometry and at the discretion of attending physician. Ten to twelve transbronchial biopsy samples are taken during each biopsy procedure. Age, sex, reason for transplant, type of transplant (single or bilateral), number of episodes of rejection in first year after transplant, highest grade of rejection, as well as the number of TBBx procedures performed were all recorded. 

### 2.2. Biopsy Evaluation

All TBBx specimens for each patient were reviewed. A pathologist blinded to all clinical data reviewed each specimen for rejection and the presence of BALT based on ISHLT criteria [7]. Routine 4 um hematoxylin-eosin sections were obtained from paraffin blocks. BALT, if present, was then immunohistochemically stained by established methods for T and B cells [8]. Specifically we stained for anti-CD4 (T Helper), anti-CD8 (cytotoxic T cells), anti-CD3 (pan T-cell marker), anti-CD45 RO (activated/memory T cells), and anti-CD20 (B cell marker) antibodies with iView DAB detection kit (Ventana medical Systems, Tucson, AZ, USA). Cells were then visually counted using Image J software (NIH, Bethesda, MD, USA) as previously described [9].

### 2.3. Data Evaluation

T- and B-cell subpopulations were expressed as cells per mm^2^. The presence of BALT and T-and B-cell population density, and group characteristics, were compared using Chi Square and Student's *t*-test, and Wilcoxon rank sum test. BALT presence and T- and B-cell subpopulations were also compared without respect to groups. Analyses were performed using Pearson correlations, Spearman rank order, and Mann-Whitney *U* test.

## 3. Results

 There were no differences in age, gender, underlying disease, and use of single- or double-lung transplant between the two groups (Table 1). There were also no significant differences in immunosuppressant medications except the multiple rejection group was given higher doses of prednisone (Table 1). Those in the multiple rejection group had significantly more rejections one year after transplant, higher grade of rejection, and more biopsies one year after transplantation (Table 2). There was also more BALT found, although this was not statistically significant, in the multiple rejection group. 

Data analyses without respect to groups displayed 40 patients with 312 TBBx procedures of which 19 (6% of procedures) displayed BALT. BALT was found as early as 26 days and as late as 644 days after lung transplantation. The presence of BALT was not associated with age, transplant type (single or bilateral), or pretransplant pulmonary diagnosis. On biopsies in which BALT was found, 12 of the 19 patients had evidence of rejection (A1 or greater), nine of which had clinically significant rejection (A2 or greater). 13 of these subjects fell into the multiple rejection group (3 or more A2 or greater rejections within the first year after transplantation), and 6 fell into the minimal rejection group (one or less A2 rejections within the first year after transplant). Of the 6 patients with BALT in the minimal rejection group 5, had no rejection in the first year after transplant, and one subject had one A1 rejection. 

 Although the presence of BALT did not appear to be associated with a clinically significant A grade rejection (A2 or greater), the finding of BALT on biopsy was associated with a greater number of rejections in the first year after transplant (*P* = 0.03). 

**Figure 1:**
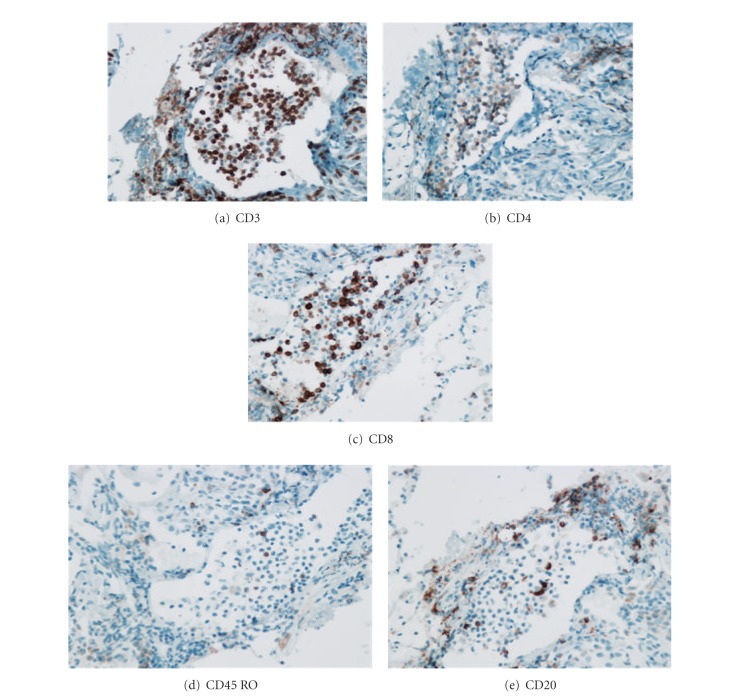
Immunohistochemical stains for subpopulations of T and B cells within BALT in a patient with multiple high-grade rejections. Patient displays a greater number of T cells and a lower CD4/CD8 ratio.

**Figure 2:**
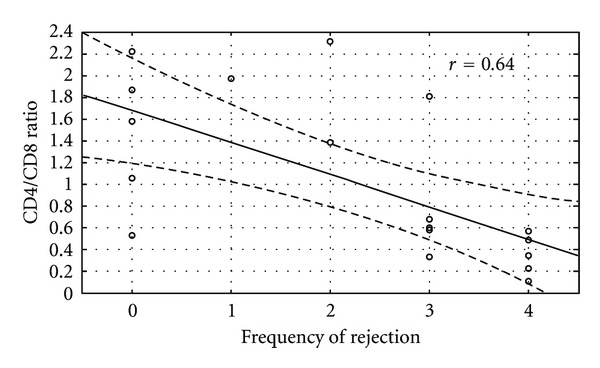
Lower CD4/CD8 ratio in patients with increased frequency of acute rejection.

**Figure 3:**
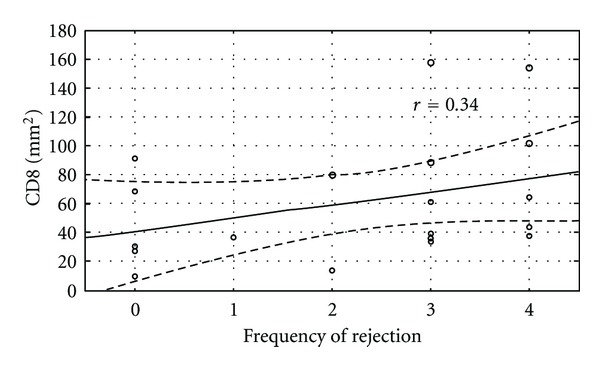
Trends towards increased density of CD8 cells in frequent acute rejection.

 The immunohistological makeup of BALT was not affected by age or transplant type. Those transplanted with COPD had an increased density of BALT (*P* = 0.005). Histologically, there were no differences in BALT cell populations between those in the multiple rejection group and those in the minimal rejection group, except a lower CD4/CD8 ratio was noted within the multiple rejection group (Table 3). The analysis showed that there were larger number of T cells compared to B cells and a larger proportion of cytotoxic T cells compared to helper T cells in those with lung rejection (Figure 1). BALT size and the density of T and B cell subpopulations did not correlate with the frequency of rejection. Comparisons between the fraction of activated T cells among all T cells (CD45 RO/CD3) showed no association with rejection frequency. However, those patients with a lower CD4/CD8 ratio had an increased frequency of rejections at on-year post lung transplant (*r* = 0.644, *P* = 0.003) (Figure 2). Those with more CD8 cells per mm^2^ to had increasing frequency of rejection at one year after transplant, although not statistically significant (*P* = 0.08) (Figure 3). 

## 4. Discussion

We report that the finding of BALT on lung biopsy is associated with more episodes of rejection at one year after lung transplantation. More than half of patients with BALT had evidence of an A grade rejection on lung biopsy. The number of rejections at one year after lung transplantation also inversely correlated with the CD4/CD8 ratio in BALT. These results are consistent with published results showing more T cells and a higher proportion of cytotoxic T cells present in BAL fluid of patients with biopsy defined rejection [10]. These results are also in agreement with animal models of lung transplant, where BALT is associated with rejection. Our results are also consistent with published data on inflammatory lung diseases, such as COPD and rheumatoid lung, where the number of BALT regions increases in conjunction with increasing disease severity (Table 4). 

However, our results seem to contradict previous data reported by Hasegawa and colleagues [5]. First we found a higher percentage of BALT present in all biopsies (6% compared to 2%). Almost half the patients in our study with BALT (47%) showed clinically significant rejection (A2 or greater) in their biopsy. The vast majority of Hasegawa's patients with BALT did not have clinically significant rejection. We showed that the presence of BALT was associated with a greater number of rejections in the first year after lung transplantation. Hasegawa did, not relate the frequency of lung rejection to the presence of BALT. He did, however, evaluate biopsies immediately prior to and after the identification of BALT and found no trend towards worsening rejection. Hasegawa also immunohistochemically stained BALT specimens. He found in all cases that CD4 cells predominated over CD8 cells. He also showed no differences between patients with low- or high-grade lung allograft rejection. Our results differ from Hasegawa, in that those in the multiple rejection group had a lower CD4/CD8 ratio. Also, a lower CD4/CD8 ratio within BALT was associated with increased frequency of acute cellular rejection. This is particularly important because the presence of BALT is rare. Even with our increased number of BALT only 6% of biopsies had BALT present, and thus the immunohistochemical makeup of BALT is likely more important than its presence. 

Our differences in results may reflect our study design. We specifically chose patients based on their frequency and severity of rejection. Thus, half the patients have number of significant episodes of rejection at one year after lung transplantation. Consequently, this group of patients had a significantly greater number of lung biopsies that were available for review, and this group likely displayed more clinical symptoms which promoted invasive diagnostic procedures. With more biopsy procedures in those with clinically significant rejection, there is a greater chance of finding BALT in those sections which displayed rejection. Also, Hasegawa reviewed biopsies for BALT at one point in time and then looked whether patients had rejection one biopsy before and one biopsy after BALT was found. Hasegawa does not mention if his subjects with BALT go on to have multiple episodes of rejection several biopsies after BALT. Thus, our study allows us to comment on, although retrospectively, on frequency of rejection and its association with BALT presence and BALT immunohistological characteristics. In contrast with previous studies, our study showed those with multiple rejections and BALT displayed a lower CD4/CD8 ratio, and the lower CD4/CD8 ratio correlated with increasing frequency of rejection. It has been suggested by Hasegawa that BALT T cells may play a role in immune tolerance. We suggest that a greater proportion of CD4 to CD8 cells may provide a protective effect. In contrast, BALT tissue containing more CD8 cells to CD4 cells shift the immune response towards greater frequency of rejection. Thus in certain patients, BALT may indicate a heightened acquired immune response that increases the risk of future rejection, while in others it may provide a protective effect. In other words, the simple presence of BALT tissue may not be diagnostic of rejection, but the presence of a lower CD4/CD8 within BALT may be indicative of rejection and also increased risk of future rejection episodes. Previous studies have shown a positive correlation between graft survival and the presence of T regulatory cell in both clinical lung transplantation and in rat models, and T regulatory cells may interfere with the development of BALT [11, 12]. The presence of regulatory T cells within BALT may provide a cellular basis for greater immune tolerance when the CD4/CD8 ratio is high, since T regulatory cells are typically CD4 positive. 

 A critical element of these types of studies is the pathological diagnosis of BALT itself. Occasionally, BALT can be confused with lymphocytic bronchiolitis. This is important to distinguish because of the association between the frequency and severity of rejection and lymphocytic bronchiolitis [13]. Though the interobserver variability for A grade rejection has been shown to be somewhat reliable, the inter-observer variability for B grade rejection (or lymphocytic bronchiolitis) is poor [14]. Data in abstract form echoes these sentiments with higher-grade lymphocytic bronchiolitis being downgraded or changed to a diagnosis of BALT by a more experienced pathologists [15]. It is clear that the expertise of the pathologist is a significant factor in not only evaluating the presence of BALT, but also in the interpretation of post transplant TBBx. The revised ISHLT criteria for lung rejection points to subtle characteristics of BALT that distinguish it from rejection-related airway inflammation [16]. It also states that BALT aggregates can expand into the fibrovascular septa, which could be confused with either perivascular, or interstitial infiltrates [16]. More focus on a clearer definition of BALT may be necessary to allow for better pathological interpretation and future research. 

 Limitations of our study are that it is retrospective in design and has a relatively small sample size so type-two error may be present. Our study design is also fundamentally different from previous studies. This could lead to bias; however, we feel that this design was optimal for hypothesis generation. We also could not control for previous infectious complications, treatment of rejection episodes, or immunosuppression therapy. Also, we used one blinded pathologist. As stated above, there is considerable disagreement on the definitions of B grade rejection and BALT itself. Nonetheless, our study shows a distinct immunohistochemical makeup of BALT in those patients with increased frequency of rejection. The link between BALT and its association with lung rejection will be better understood with a firmer pathological definition of BALT and prospective studies involving immunohistochemical analysis of leukocyte subpopulations with relevant functional activities, including T regulatory cells. These further studies may allow for an improved diagnosis of those at risk for multiple rejections.

## Figures and Tables

**Table 1:** Demographics.

	Minimal rejection	Multiple rejection	*P* value
	*n* = 20	*n* = 20	
Age, yrs	52.7 ± 2.05	55.49.7 ± 2.26	ns
Sex	F = 11	F = 8	ns
Disease requiring transplant	COPD = 10	COPD = 13	ns
	IPF = 5	IPF = 5	
	Other = 5	Others = 2	
Transplant Type (Bilateral %)	65%	40%	ns
Tacrolimus %	45%	45%	ns
Mycophenolate %	95%	90%	ns
Prednisone dose (mg)/day	14.0 ± 1.4	19.7 ± 0.7	<0.005

F: female, COPD: chronic obstructive pulmonary disease, IPF: idiopathic pulmonary fibrosis, and others: pulmonary hypertension and bronchiectasis.

**Table 2:** Biopsy results.

	Minimal rejection	Multiple rejection	*P* value
	*n* = 20	*n* = 20	
Number of biopsies/patient^∗^	6.7 ± 0.5	8.9 ± 0.6	<0.05
Average number rejections/patient^∗^	0.35 ± 0.13	3.2 ± 0.3	<0.005
Highest grade of rejection^∗^	0.35 ± 0.13	2.4 ± 0.11	<0.005
BALT present (yes)	6	13	ns

^∗^ One year after transplant.

**Table 3:** Cellular characteristics.

	Minimal rejection	Multiple rejection	*P* value
CD4/mm^2^	53.05 ± 8.82	46.91 ± 12.37	0.31
CD8/mm^2^	43.7 ± 12.38	69.8 ± 12.6	0.17
CD4/CD8	1.53 ± 0.26	0.77 ± 0.18	0.04
CD3/mm^2^	99.58 ± 18.9	137.39 ± 23.65	0.76
CD20/mm^2^	41.26 ± 16.05	55.12 ± 18.90	0.89
CD3/CD20	3.15 ± 0.62	3.94 ± 0.77	0.57
CD45 RO/mm^2^	21.41 ± 11.4	43.8 ± 14.12	0.17
CD45 RO/CD3	0.19 ± 0.06	0.29 ± 0.05	0.14

**Table 4:** Summary of recent BALT literature.

Author	Disease state	BALT conclusion
Hogg et al., 2004 [2]	COPD	Increased BALT presence with worsening disease severity
Rangel-Moreno et al., 2006 [3]	Rheumatoid lung	Induced BALT presence correlates with increased lung tissue damage
Prop et al., 1985 [4]	Animal lung transplant	Presence of BALT correlates with rejection. Removal of BALT through irradiation of donor abrogates rejection
Hasegawa et al., 1999 [5]	Human lung transplant	BALT presence correlates with minimal to no rejection. BALT may play role in immune tolerance
